# Safe Transition from Open to Total Minimally Invasive Esophagectomy for Cancer Utilizing Process Management Methodology

**DOI:** 10.3390/jcm13154364

**Published:** 2024-07-26

**Authors:** Milos Bjelovic, Dragan Gunjic, Tamara Babic, Milan Veselinovic, Marija Djukanovic, Dario Potkonjak, Vladimir Milosavljevic

**Affiliations:** 1 Euromedic General Hospital, Bulevar umetnosti 29, 11070 Belgrade, Serbia; drgunja@gmail.com; 2 School of Medicine Foca, University East Sarajevo, Studentska 5, 73300 Foca, Bosnia and Herzegovina; 3 Department for Minimally Invasive Upper Digestive Surgery, Hospital for Digestive Surgery, Clinical Center of Serbia, Dr Koste Todorovica Street 6, 11000 Belgrade, Serbia; b.tamara86@gmail.com (T.B.); m_veselinovic@yahoo.com (M.V.); dariopotkonjak10@gmail.com (D.P.); 4 School of Medicine, University of Belgrade, Dr Subotica Street 8, 11000 Belgrade, Serbia; 5 Department of Anesthesiology and Resuscitation, Hospital for Digestive Surgery, Clinical Center of Serbia, Dr Koste Todorovica Street 6, 11000 Belgrade, Serbia; djukanovic_marija@yahoo.com; 6 University Hospital Medical Center Bezanijska Kosa, Dr Zorza Matea Street, 11000 Belgrade, Serbia; milosavljevicvladimir10@gmail.com

**Keywords:** esophageal cancer, surgery, minimally invasive esophagectomy, process management

## Abstract

**Background:** The global shift from open esophagectomy (OE) to minimally invasive esophagectomy (MIE) for treating esophageal cancer is well-established. Recent data indicate that transitioning from hybrid minimally invasive esophagectomy (hMIE) to total minimally invasive esophagectomy (tMIE) can be challenging due to concerns about higher leakage rates and lower lymph node counts, especially at the beginning of the learning curve. This study aimed to demonstrate that a safe transition from OE to tMIE for cancer is possible using process management methodology. Methods: A step-change approach was adopted in process management planning, with hMIE serving as an intermediate step between OE and tMIE. This single-center, case–control study included 150 patients who underwent the Ivor Lewis procedure with curative intent for esophageal cancer. Among these patients, 50 underwent OE, 50 hMIE (laparoscopic procedure followed by conventional right thoracotomy), and 50 tMIE (laparoscopic and thoracoscopic approach). A preceptored training scheme was implemented during execution, and treatment results were monitored and controlled to ensure a safe transition. Results: During the transition, the tMIE group was not worse than the hMIE and OE groups regarding operation duration (*p* = 0.135), overall postoperative complications (*p* = 0.020), anastomotic leakage rates (*p* = 0.773), 30-day mortality (*p* = 1.0), and oncological outcomes (based on R status (*p* = 0.628) and 2-year survival (*p* = 0.967)). Additionally, the tMIE group showed superior results in terms of major postoperative pulmonary complications (*p* = 0.004) and ICU stay duration (*p* < 0.001). **Conclusions:** Utilizing managerial methodology and practice in surgery, as a bridge between interdisciplinary and transdisciplinary approaches, demonstrated that transitioning from OE to tMIE, with hMIE as an intermediate step, is safe and feasible without compromising outcomes.

## 1. Introduction

Despite advancements in preoperative and postoperative care, esophagectomy remains a highly invasive procedure involving considerable morbidity and mortality [1]. The global shift from open esophagectomy (OE) to minimally invasive esophagectomy (MIE) in treating esophageal cancer is well-established [2]. Within the scope of MIE, various procedures exist, with the most commonly used being hybrid minimally invasive esophagectomy (hMIE) and total minimally invasive esophagectomy (tMIE) [3].

While the benefits of the minimally invasive esophagectomy (MIE) approach compared to open esophagectomy (OE) are undeniably clear, there remains an open question as to whether hybrid minimally invasive esophagectomy (hMIE) can meet the expectations of the minimally invasive (MI) approach and improve treatment outcomes compared to OE, or if it is merely a transitional step towards total minimally invasive esophagectomy (tMIE. Recent observational trial results published by Petel et al. indicate that tMIE is associated with fewer pulmonary infective and overall postoperative complications compared to hMIE for resectable esophageal and gastroesophageal junction (GEJ) cancer [4]. Additionally, a propensity score-matched trial by Berlth et al. concluded that tMIE is superior to hMIE in terms of postoperative pain and pneumonia rates [5]. Conversely, a recently published network meta-analysis by Siaw-Acheampong et al. confirmed that minimally invasive techniques for esophagectomy are associated with reduced perioperative morbidity and shorter hospital stays, without compromising oncological outcomes. However, no single approach has demonstrated clear overall superiority [6].

In relation to estimating and analyzing trends and projections, most discussions and conclusions tend to be projections of the current system, heavily reliant on the assumptions used in calculations. In relation to the future implications of this type of work, in surgery, an increasing number of surgeons are adopting newer techniques. In the Western world, robot-assisted minimally invasive esophagectomy/surgery (RAMI) is becoming more common. Currently, the main issue with RAMI is its lack of cost-effectiveness compared to laparoscopic and thoracoscopic approaches. However, with several new robots expected to enter the market soon, the surgical community is poised to enter a new era of minimally invasive surgery (MIS)—the era of RAMIS. It is unlikely that the hybrid concept will survive in this new era.

Finally, understanding and meeting patient expectations is a crucial goal of treatment. There is no doubt that less invasive treatments are more appreciated by patients.

Transitioning from open to minimally invasive surgery raises specific concerns for both patients and surgeons. Patients worry about the effectiveness of MIS in fully addressing their condition and fear increased cancer recurrence due to fewer lymph nodes being examined. They also have anxiety about potential complications such as higher leakage rates and the overall safety of the new technique. Surgeons face concerns over the technical challenges of MIS, the steep learning curve, and the potential for decreased lymph node counts affecting treatment decisions. Both parties are apprehensive about ensuring consistent and high-quality outcomes during this transition. Implementing a minimally invasive esophagectomy (MIE) program is technically demanding [7]. Recent data confirmed that transitioning from hybrid minimally invasive esophagectomy (hMIE) to total minimally invasive esophagectomy (tMIE) can be challenging, primarily due to concerns about higher anastomotic leakage (AL) rates and lower lymph node counts, particularly at the beginning of the learning curve [8]. However, a growing number of studies support the view that the shift from hybrid to total MIE is both safe and feasible, without compromising outcomes [9,10,11].

Process management is defined as the application of knowledge, skills, tools, and techniques to meet project requirements [12]. Essentially, it involves the processes necessary to guide a team from point A (open esophagectomy, OE) to point B (total minimally invasive esophagectomy, tMIE). To achieve this, the project team must manage processes within the following five main stages: initiation, planning, execution, monitoring and controlling, and closeout [13]. Activities during the initiation phase include project initiation meetings, identifying the project team, developing the necessary resources for the project plan, and establishing the project management infrastructure. Process management methodology is particularly pertinent to the transition from open to minimally invasive surgery because it systematically addresses the increased complexity and potential risks involved. It ensures consistency and high-quality outcomes by standardizing protocols and implementing quality control measures. The methodology also facilitates effective training and skill development, helping surgeons and staff quickly adapt to new techniques. Overall, it provides a structured framework that ensures a smooth, efficient shift to MIS, addressing both technical and operational challenges.

This paper aims to demonstrate that a safe transition from OE to tMIE for cancer is achievable through the application of process management methodology.

## 2. Materials and Methods

Historically, planning methodologies focused on incremental and continuous improvements in work processes. Recently, however, organizations have increasingly adopted step-change strategies that leverage new technologies and accumulated knowledge to achieve significant improvements in effectiveness and efficiency. In this project, a step-change philosophy was adopted, using hMIE as an intermediate step between OE and tMIE. Therefore, the standard of care evolved from OE before 2009, to hMIE between 2009 and 2013, and to tMIE after 2013.

All subjects gave informed consent before study enrollment. The study was reviewed and approved by the Clinical Center of Serbia Institutional Review Board (decision number 1575/15).

In the execution phase, this single-center study included a total of 150 patients selected through convenience sampling. These patients underwent the Ivor Lewis procedure with curative intent for middle- and lower-third esophageal cancer (either squamous carcinoma or adenocarcinoma) between January 2007 and June 2019.

All patients underwent preoperative workup which included symptom evaluation, barium swallow radiography, upper flexible endoscopy with biopsy, computed tomography (CT) of the thorax and abdomen, pulmonary evaluation (flexible bronchoscopy and pulmonary function tests), and positron emission tomography (PET) when appropriate. Patients with locally advanced tumors received neoadjuvant therapy followed by surgery. Before surgery, all patients underwent multidisciplinary team consultation with a diagnostic and therapeutic workout according to the European Society for Medical Oncology (ESMO) recommendations [14]. Assessment of functional, nutritional, and comorbidity status was performed before surgery. Patients with other synchronous or metachronous neoplasms, confirmed metastatic disease, histology other than a squamous cell carcinoma or adenocarcinoma of the esophagus, or poor general status with severe co-morbidities were excluded from the study.

Out of 150 patients, 50 underwent OE, 50 hMIE (laparoscopic procedure followed by conventional right thoracotomy) and 50 patients underwent tMIE (laparoscopic and thoracoscopic approach). Preoperative data did not influence the operative approach. Subsequent comparative analysis of the preoperative data did not indicate selection bias or potential confounding. The three groups were compared in respect of perioperative data, as well as postoperative course details.

All procedures were performed by the same surgical team, experienced in esophageal and minimally invasive surgery, using a standardized operative technique. The abdominal part of the procedure included gastric mobilization, narrow gastric tube formation (5–6 cm wide gastric tube), and lymph node dissection. To reduce tension to the transplant and the anastomosis, Kocher’s maneuver was a standard part of the procedure. The thoracic part of the procedure was performed with the patient positioned in left lateral decubitus with blockade of the right lung (single lumen tube). Patient position and trocar placement for the MI approach (both laparoscopic and thoracoscopic) were adopted from Luketich et al. [15]. After subtotal esophagectomy and two-field standard lymph node dissection, gastric pull-up was performed. Intrathoracic esophago–gastro anastomosis was created in the upper mediastinum and fully covered with an omental flap preserved along the greater curvature of the stomach. A nasogastric tube, for gastric decompression, was placed in the gastric conduit under direct vision and removed within 48 h after surgery. Pleural tenting was a standard part of the surgical procedure.

The specimen assessment was conducted by specified pathologists, according to the 8th edition of the American Joint Committee on Cancer protocol from 2017 [16]. Furthermore, the number of the harvested lymph nodes and the R status were assessed as key features of the oncological outcomes of the methods used.

All patients underwent antibiotic and deep venous thrombosis prophylaxis as well as respiratory physiotherapy and early mobilization. A contrast barium meal was routinely performed on the fifth postoperative day, followed by a clear liquid diet.

After hospital discharge, the first check-up was performed a month after surgery, then at three to four-month intervals for the first year, followed by six-monthly reviews during the second year, and annually thereafter [17].

In this case–control study, perioperative and postoperative data for the patients treated with the MI approach (hMIE and tMIE) were collected, monitored, and controlled from a prospectively developed database. The OE group was a historical cohort. Complications in the perioperative course were defined and classified according to the international consensus on standardization of data collection for complications associated with esophagectomy, graded according to the Clavien–Dindo system, and calculated utilizing the Comprehensive Complication Index (CCI) [18,19,20]. Oncological results were evaluated by analyzing a number of harvested lymph nodes, surgical margins (R status), and overall survival two years after surgery.

Primary endpoints in monitoring and controlling were these safety endpoints: non-inferiority of the MIE groups, compared to OE group, in the onset of

- total number of and the most significant early postoperative complications, i.e., anastomotic leakage (AL), major postoperative pulmonary complications (MPPC), etc.
- significant postoperative complications classified according to Clavien–Dindo classification ≥ II
- complications grade according to the Comprehensive Complication Index (CCI)
- 30-d mortality rates [19,20]

Secondary endpoints were efficacy endpoints, including these perioperative characteristics and oncological outcomes:

- non-inferiority of the MIE groups, compared to OE group, in respect to the duration of the operation and blood loss/transfusion
- reduction in MIE groups, compared to OE group, in the ICU and overall hospital stay
- non-inferiority of the oncological outcomes of the MIE groups, compared to the OE group, based on the number of harvested lymph nodes, R status, and short-term survival.

Monitoring and controlling the treatment results concerning safety and efficacy was a continuous process, with the aim to modify the practice to achieve optimal project/treatment results.

### Statistical Analysis

Numerical data are presented as the arithmetic mean and standard deviation or median with range (minimum–maximum), depending on the distribution. Mathematical (coefficient of variation, skewness and kurtosis, Kolmogorov–Smirnov and Shapiro–Wilk tests) and graphical (histogram, box plot, Q-Q diagram) criteria for normal distribution were used. Categorical data are presented as absolute and relative numbers. The three study groups were compared using one-way ANOVA with Tukey post-hoc testing or the Kruskal–Wallis test followed by the Mann–Whitney test if data were numerical, whilst for categorical data, Chi-square or Fisher’s exact test were applied. Survival rates were evaluated and graphically presented using the Kaplan–Meier method, with the log-rank test for comparing groups. Cox regression was the method of choice for analyzing predictors for mortality in each study group separately. Multivariate models with regression coefficient B, hazard ratio (HR), 95% confidence interval of the hazard ratio (95%CI HR) and *p* value are presented. They were constructed of significant variables from univariate analysis. The backward Wald method was used, and only the first and last steps were reported. The VIF method was used to analyze collinearity (all variables with VIF ≥ 5 were excluded from the multivariable model). All statistical results were considered significant if *p* ≤ 0.05. For the total number of 150 patients (divided into 3 study groups), the minimal detectable hazard ratio of median survival of 1.8, accrual interval of 11 years, follow-up of 2 years and obtained median time to failure (defined as death) in the group with the smallest time to failure, and the power of the study was 90%. All statistical analysis was performed using SPSS Statistics for Windows, Version 21.0. (IBM Corp, Armonk, NY, USA, released 2012).

During the end of the project, project documents were checked, and the final few items in datasheets and statistical analysis were completed. The results were discussed by the project team.

## 3. Results

The analyzed groups were homogenous in respect to average patients’ age, sex, body mass index (BMI), and American Society of Anesthesiologists (ASA) score, as well as the stage of the disease (Table 1). However, study groups differed in respect to the tumor localization (*p* = 0.033) and histological type of the tumor (*p* = 0.001). Squamose cell carcinoma (SCC) was the most prevalent histological type in the OE group, while esophageal adenocarcinoma (EAC) was the most prevalent type in the tMIE group. SCC and EAC were almost equally distributed in the hMIE group (Table 1).

The frequency of postoperative complications was highest in the OE group (Table 2). They were significantly more frequent in the OE group when compared to both the hMIE and tMIE groups (*p* = 0.024 and 0.013, respectively). In addition, the transition from hMIE to tMIE did not influence an increase in the prevalence of postoperative complications (*p* = 0.822).

Similarly, major postoperative pulmonary complications were also most common in the OE group. They were significantly more frequent in the OE group than in the tMIE group (*p* < 0.001) with no difference between the OE and hMIE groups, indicating that a minimally invasive thoracic approach could positively influence MPPC rates. One patient in the OE group, one in hMIE, and two patients in the tMIE group had anastomotic leakage, graded as ≥type 2 leakage. Frequencies of this specific complication were relatively low, with no statistically significant difference between the groups (*p* = 0.773). Nevertheless, this suggests that a safe transition to tMIE could be achieved without drawbacks.

After analyzing complications according to the Clavien–Dindo classification (CDC), no statistically significant difference in distribution between the groups was observed (*p* = 0.815), with more than 50% of complications graded as a CDC grade II. On the contrary, the CCI score was significantly higher in the OE group compared to both the hMIE and tMIE groups (*p* = 0.048 and *p* = 0.017, respectively), indicating that in the OE group, one patient tended to have more than one potentially serious postoperative complication. The overall 30-day mortality rate was 1.3%, with no significant differences between the groups.

During the transition, the tMIE group was not worse than the hMIE and OE groups in respect of the duration of the operation (*p* = 0.135) (Table 3).

Moreover, the two MIE groups were superior compared to the OE group in respect to blood transfusion (*p* = 0.005) and the number of harvested lymph nodes (*p* = 0.001). Blood transfusion was greatest in the OE group (med = 275 mL), with no difference between the hMIE and tMIE groups (*p* = 0.180). Although the number of retrieved lymph nodes was oncologically adequate in all groups, the median number was significantly higher in the hMIE group (31.5) in comparison with tMIE and OE groups (28.5 and 26, respectively), with no significant difference between the OE and tMIE groups (*p* = 0.103).

The duration of ICU stay was longest in the OE group in comparison with the other two groups (*p* < 0.001), and it was significantly longer in the hMIE group compared to the tMIE group (*p* < 0.001), indicating faster early recovery after the least invasive approach (Table 2). The mean duration of hospital stay after OE surgery was statistically longer than after the hMIE or tMIE approaches There was no statistically significant difference in duration of hospitalization between the hMIE and tMIE groups (*p* = 0.838)

**Figure 1:**
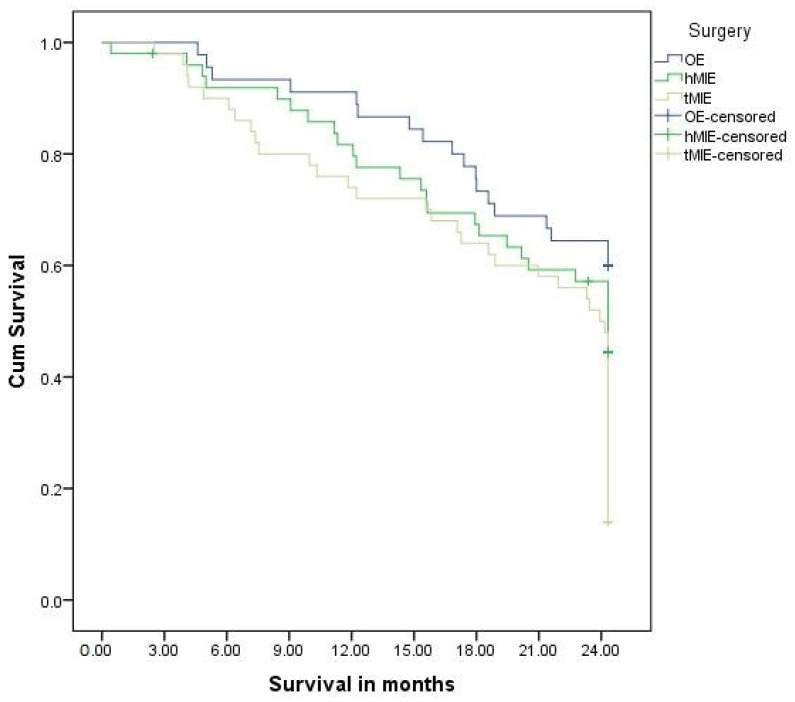
Average survival time of patients of the two groups in months.

Positive and distal resection margins came out to be negative in 94% of patients in the OE group, 95.9% of patients in the hMIE group, and 96% patients in the tMIE group (*p* = 0.628). Mean survival in the OE group was 20.73 (se = 0.85) months, in hMIE was 19.02 (se = 1.04) months and in tMIE was 18.73 (se = 1.08) months, with no significant differences between the groups (Figure 1).

## 4. Discussion

The utilization of managerial methodology and practice in surgery serves as a bridge between interdisciplinary and transdisciplinary approaches [21]. Project management focuses on the processes and activities necessary to complete a project, such as implementing a new surgical procedure. In this paper, we emphasize project management using the step-change concept, with hMIE as an intermediate step between OE and tMIE. Leadership is crucial, not only during the initiation and planning phases but throughout the entire management process.

Process management in surgery is applied to preoperative, intraoperative, and postoperative procedures. The development and implementation of evidence-based treatment pathways help standardize and structure the treatment process in the surgical department and operating rooms. Additionally, process management plays a significant role in the overall provision of health care and services [22].

During execution, adhering to a standardized operative technique is crucial, due to the numerous technical challenges involved in transitioning between different approaches. Therefore, minimizing variability is essential.

Process management plays a vital role in optimizing capacity while prioritizing maximum patient safety and employee satisfaction. An exemplary case study demonstrates the benefits of process management, showing how innovative approaches can increase capacity while simultaneously reducing costs [22].

Laparoscopic and thoracoscopic esophageal surgery is intricate and requires specialized training to develop advanced skills in both laparoscopy and thoracoscopy. A modular training system has proven effective in minimally invasive prostatectomy and has subsequently been applied in other areas of minimally invasive surgery (MIS), allowing trainees to systematically undertake specific parts of operations in a planned and structured manner [11,23,24].

Essentially, the preceptored training scheme for transitioning from open to MIS includes several key elements. It starts with a comprehensive theoretical module covering the principles and techniques of MIS. This is followed by hands-on simulations and lab sessions, focusing on dexterity and precision. The final phase consists of supervised surgeries, where trainees assist and gradually take on more responsibility under the guidance of experienced MIS surgeons. To pass the training scheme, trainees must demonstrate proficiency in simulations, complete a minimum of ten supervised MIS procedures, and receive positive evaluations from their preceptors on both technical skills and adherence to safety protocols. In our case, all procedures were performed by the same surgical team, experienced in esophageal and advanced UGI minimally invasive surgery. Thus, preceptored training focused on the surgical element, involving the performance of specific parts of larger cases during other procedures as well. For the laparoscopic part of the procedure, three distinct training modules were identified. The first module involved patient setup and positioning, creation of a pneumoperitoneum, port placement, and gastric and duodenal mobilization (Kocher’s maneuver). The second module focused on lymph node dissection along the coeliac region. The third module covered gastric tube formation. Given that the Department for Minimally Invasive Upper Digestive Surgery is part of the Division for Esophageal and Gastric Surgery, comprehensive training encompassed all three modules during other advanced minimally invasive (MI) procedures. Specifically, the first step was practiced during laparoscopic hiatal hernia repair (especially giant PEH repair) and laparoscopic myotomy for achalasia. The second step was integrated into laparoscopic gastrectomy for cancer, and the third step was refined through laparoscopic bariatric procedures for morbid obesity, notably laparoscopic sleeve gastrectomy. The transition from hMIE to tMIE was meticulously planned, following sufficient experience with the laparoscopic hybrid procedure.

Similarly, in the preceptored training scheme for the thoracoscopic part of the procedure, two distinct training modules were identified. The first module included patient setup and positioning, port placement, and esophageal mobilization with lymph node dissection in the posterior mediastinum. The second module focused on creating an intrathoracic esophago–gastro anastomosis in the upper mediastinum. These components were also practiced extensively during other advanced minimally invasive (MI) procedures, such as thoracoscopic excision of esophageal leiomyoma or diverticula, esophageal myotomy for diffuse esophageal spasm, and anastomosis formation during laparoscopic total gastrectomy for cancer.

Mastering suturing and intracorporeal knotting skills is vital for ensuring the safety of minimally invasive (MI) surgery. In the realm of esophagogastric anastomosis techniques, hand-sewn intrathoracic anastomosis is particularly favored in the era of robot-assisted minimally invasive surgery (RAMIS). Looking forward, anticipating future advancements is crucial as they will profoundly influence the professional landscape. Above all, maintaining unwavering standards of surgical quality is essential, regardless of the chosen approach.

Regarding the monitoring and control of execution, preoperative data did not dictate the operative approach. Comparative analysis of preoperative data did not reveal selection bias or significant confounding factors, except for variations in tumor location and histological type. Globally, there has been a decline in the incidence of esophageal squamous cell carcinoma (SCC) since 1986, whereas both esophageal adenocarcinoma (EAC) and gastroesophageal junction (GEJ) cancer have seen marked increases. Furthermore, the 2017 revision of the UICC classification reclassified GEJ adenocarcinoma, particularly Siewert type II cancer, as esophageal cancer. This reclassification has led to a notable rise in the number of esophagectomies performed worldwide. In our practice, over the past decade, EAC and GEJ adenocarcinoma have become the most prevalent indications for esophagectomy.

In our study, the incidence of postoperative complications was highest in the OE group. Complications were significantly more frequent in the OE group compared to both the hMIE and tMIE groups. However, there was no significant difference between the hMIE and tMIE groups, indicating that transitioning from hMIE to tMIE did not lead to an increase in postoperative complications. Similar findings were reported by Berlth et al. and Bonavina et al. [5,25].

Major postoperative pulmonary complications (MPPCs) are the main contributor to postoperative morbidity after esophagectomy, accounting for up to 31% of all postoperative complications [26]. In our study, MPPCs were most common in the OE group. They were significantly more frequent in the OE group than in the tMIE group, suggesting that a minimally invasive thoracic approach could reduce MPPC rates. The MIRO trial noted that pulmonary complications constituted a significant portion of postoperative issues, with rates of 18% in the hMIE group and 30% in the OE group [27]. In the TIME trial, tMIE was associated with a lower incidence of pulmonary infections (9%) compared to the OE group (29%) [28]. Patel et al. concluded that tMIE is linked to fewer pulmonary infections compared to hMIE [4]. Berlth et al. also found a significant reduction in postoperative pneumonia after tMIE compared to hMIE, although overall postoperative complication rates did not differ significantly [5]. The exact mechanism by which the hybrid approach in the MIRO trial achieved complication rates comparable to full minimally invasive procedures remains unclear and challenging to ascertain. Nevertheless, published studies consistently highlight the thoracic phase as a critical factor influencing postoperative outcomes following esophagectomy [29].

One of the primary concerns regarding tMIE is the technical challenges associated with performing safe thoracoscopic esophagogastric anastomosis [8]. Despite various techniques and modifications, there is no consensus on which anastomotic method yields the best patient outcomes [30]. In our study, esophagogastric anastomosis was performed in the chest using a standardized technique of circular stapled anastomosis. We found no statistically significant difference in anastomotic leak (AL) rates between the OE, hMIE, and tMIE groups (*p* = 0.773). The prevalence of AL was 2% in the OE and hMIE groups, and 4% in the tMIE group.

Introducing a new procedure must not unnecessarily prolong operations or disrupt hospital services in a way that compromises patient care or violates cancer care targets. We believe that the learning curve effects were minimal in our study due to extensive experience in other advanced upper gastrointestinal laparoscopic and thoracoscopic procedures, with the same surgical team performing all operations. Similar findings were reported by Grimminger et al. [9].

In our study, the average ICU stay was shorter in the tMIE group compared to the OE and hMIE groups, indicating quicker early recovery with the least invasive approach. Pooled data from 282 patients across six European centers showed 30-day and in-hospital mortality rates of 2.1% [31]. Similarly, our study found no significant differences in mortality rates among the observed groups.

In terms of oncological outcomes, the number of retrieved lymph nodes was sufficient across all groups, with higher counts in the hMIE and tMIE groups compared to OE [32]. A recent data-mining study found that 10% of esophagectomy patients had positive resection margins [33]. In our trial, negative proximal and distal resection margins were observed in 94% of the OE group, 95.9% of the hMIE group, and 96% of the tMIE group.

There was no statistically significant difference in 2-year survival rates between the groups when stratified by disease stage. This trial adds to a limited body of evidence suggesting that tMIE can be performed safely and effectively without compromise [11,34].

This research is pivotal in the field because it fills the knowledge gap of how structured process management can enhance surgical outcomes and patient safety during this transition. By demonstrating the effectiveness of standardized protocols, risk management, and continuous improvement, the study provides a blueprint for best practices in adopting MIS. Its conclusions could lead to more consistent, high-quality surgical results, reducing complications and improving patient recovery times. Ultimately, this research has the potential to revolutionize therapeutic practices by making MIS a safer and more reliable option for a broader range of patients.

One of the limitations of this trial is its single-center design. Additional limitations include the use of a historical cohort as a comparator, highlighting the need for prospective randomized trials to validate the findings. However, conducting such a study could raise ethical concerns. A technical limitation of this approach is that it represents just one of many potential strategies for adopting change. In our organizational schema, general and digestive surgeons perform esophageal surgery. Therefore, beginning with a laparoscopic hybrid approach was a logical choice. Conversely, thoracic surgeons might approach this differently. Ultimately, the emphasis lies on the concept and methodology rather than the specific execution.

Despite these limitations, this analysis serves as a foundational step for future research directions, which may involve larger multicenter trials, longer follow-up periods, and investigations into the role of robotic-assisted minimally invasive surgery (RAMI).

## 5. Conclusions

Total minimally invasive esophagectomy (tMIE), currently the least invasive procedure available, has the potential to enhance treatment specificity and improve patient outcomes and experiences, particularly regarding major postoperative pulmonary complications (MPPC) and ICU stay duration. While there are numerous challenges in implementing this treatment approach for eligible patients, the progress thus far suggests that tMIE may gradually become the standard of care.

Our objective is to ensure that the transition from more invasive treatment modalities to tMIE is as safe as possible. In this regard, employing process management methodologies, with hMIE serving as an intermediary step, can significantly enhance the safety and effectiveness of this transition.

## Figures and Tables

**Table 1:** Preoperative characteristics.

Preoperative Characteristics	OE (n = 50)	hMIE (n = 50)	tMIE (n = 50)	*p* ^a^	*p* ^b^	*p* ^c^	*p* ^d^
Age (years), mean ± sd	59.20 ± 9.23	62.92 ± 9.81	61.34 ± 10.52	0.170	0.147	0.525	0.703
Gender, n male/female	41/9	42/8	43/7	0.862	0.790	0.585	0.779
ASA score, mean ± sd	2.28 ± 0.58	2.38 ± 0.63	2.08 ± 0.90	0.111	0.760	0.374	0.098
ASA grade, n (%)							
ASA grade 0	0	0	4 (8.0)				
ASA grade I	2 (4.3)	1 (2.0)	4 (8.0)	0.190	0.730	0.281	0.170
ASA grade II	31 (66.0)	32 (64.0)	28 (56.0)				
ASA grade III	13 (27.7)	14 (28.0)	12 (24.0)				
ASA grade IV	1 (2.1)	3 (6.0)	2 (4.0)				
BMI (kg/m^2^), mean ± sd	23.89 ± 3.62	22.99 ± 3.93	24.98 ± 5.17	0.079	0.589	0.455	0.063
Preoperative albumins (g/L), mean ± sd	41.05 ± 4.81	40.27 ± 4.38	40.25 ± 5.15	0.760	0.963	0.987	1.000
Smoking, n (%)	22 (44.0)	23 (52.3)	24 (68.6)	0.081	0.423	0.025	0.143
DLCO (%), mean ± sd	77.45 ± 8.86	72.94 ± 10.77	73.22 ± 9.73	0.070	0.100	0.175	0.993
Tumor localization, n Middle-third/ Lower-third	21/29	16/34	9/41	0.033	0.300	0.009	0.106
Histological subtype, n SCC/Adenocarcinoma	31/19	24/26	13/37	0.001	0.159	<0.001	0.023
TNM classification, n (%)							
T0	1 (2.0)	1 (2.0)	1 (2.0)	0.132	0.073	0.273	0.262
T1	12 (24.0)	7 (14.0)	5 (10.0)				
T2	4 (8.0)	1 (2.0)	6 (12.0)				
T3	32 (64.0)	33 (66.0)	34 (68.0)				
T4	1 (2.0)	8 (16.0)	4 (8.0)				
N0	15 (30.0)	16 (32.0)	11 (22.0)	0.651	0.435	0.780	0.442
N1	15 (30.0)	10 (20.0)	15 (30.0)				
N2	11 (22.0)	9 (18.0)	12 (24.0)				
N3	9 (18.0)	15 (30.0)	12 (24.0)				
M0	50 (100.0)	50 (100.0)	49 (98.0)	1.000	N/A	1.000	1.000
M1	0 (0.0)	0 (0.0)	1 (2.0)				
Preoperative Hgb (g/L), mean ± sd	128.60 ± 15.01	131.48 ± 17.93	134.00 ± 22.7	0.643	0.767	0.692	0.927

^a^ Comparison between OE, hMIE and tMIE, ^b^ Comparison between OE and hMIE, ^c^ Comparison between OE and tMIE, ^d^ Comparison between hMIE and tMIE according to one-way ANOVA with Tukey post-hoc testing or Kruskal-Wallis with Mann-Whitney test for numerical and Chi-square or Fisher’s exact test for categorical data.

**Table 2:** Postoperative characteristics.

Postoperative Characteristics	OE (n = 50)	hMIE (n = 50)	tMIE (n = 50)	*p* ^a^	*p* ^b^	*p* ^c^	*p* ^d^
Postoperative complications, n (%)	25 (50.0)	14 (28.0)	13 (26.0)	0.020	0.024	0.013	0.822
Major pulmonary complications, n (%)	20 (40.0)	11 (22.0)	6 (12.0)	0.004	0.052	0.001	0.183
Anastomotic leakage, n (%)	1 (2.0)	1 (2.0)	2 (4.0)	0.773	1.000	0.558	0.558
Reinterventions, n (%)	1 (2.0)	3 (6.0)	1 (2.0)	0.437	0.307	1.000	0.307
CCI, med (min–max)	10.45 (0–100)	0 (0–100)	0 (0–33.7)	0.032	0.048	0.017	0.720
Dindo-Clavien grade, n (%)							
II	17 (68.0)	7 (50.0)	8 (61.5)	0.815	0.182	0.105	1.000
III	6 (24.0)	5 (35.7)	5 (38.5)				
IV	1 (4.0)	1 (7.1)	0 (0.0)				
V	1 (4.0)	1 (7.1)	0 (0.0)				
ICU stay (days), med (min–max)	4 (1–21)	2 (1–11)	1 (1–10)	<0.001	<0.001	<0.001	<0.001
Length of hospitalization (days), med (min–max)	15 (1–48)	13 (2–45)	13 (8–28)	0.009	0.012	0.005	0.838
R status, n (%)							
0	47 (94.0)	47 (95.9)	48 (96.0)	0.628	0.617	0.617	1.000
R1	3 (6.0)	1 (2.0)	1 (2.0)				
R2	0 (0.0)	1 (2.0)	1 (2.0)				
30 days mortality	1 (2.0)	1 (2.0)	0 (0)	1.000	1.000	1.000	1.000

^a^ Comparison between OE, hMIE and tMIE, ^b^ Comparison between OE and hMIE, ^c^ Comparison between OE and tMIE, ^d^ Comparison between hMIE and tMIE According to One way ANOVA with Tucky post hoc testing or Kruskal-Wallis with Mann-Whitney test for numerical and Chi-square or Fisher’s exact test for categorical data.

**Table 3:** Intraoperative characteristics.

Intraoperative Characteristics	OE (n = 50)	hMIE (n = 50)	tMIE (n = 50)	*p* ^a^	*p* ^b^	*p* ^c^	*p* ^d^
Duration of operation (min), mean ± sd	339.78 ± 25.27	327.77 ± 37.40	327.42 ± 58.4	0.135	0.371	0.778	0.123
Conversion, n (%)	0 (0)	2 (4.0)	1 (2.0)	0.360	0.153	0.315	0.558
Blood transfusion (ml), med (min–max)	275.0 (0–1125)	0 (0–1250)	0 (0–795)	0.005	0.032	0.004	0.180
Number of harvested lymph nodes, med (min–max)	26 (14–56)	31.50 (20–59)	28.50 (7–59)	0.001	<0.001	0.103	0.022
Positive lymph nodes, med (min–max)	2.5 (0–28)	2.5 (0–38)	2 (0–26)	0.990	0.888	0.981	0.917
Disease stage, n (%)							
0	1 (2.0)	1 (2.0)	1 (2.0)	0.206	0.180	0.059	0.720
I	10 (20.0)	9 (18.0)	6 (12.0)				
II	5 (10.0)	9 (18.0)	6 (12.0)				
III	32 (64.0)	23 (46.0)	25 (50.0)				
IV	2 (4.0)	8 (16.0)	12 (24.0)				

^a^ Comparison between OE, hMIE and tMIE, ^b^ Comparison between OE and hMIE, ^c^ Comparison between OE and tMIE, ^d^ Comparison between hMIE and tMIE according to one-way ANOVA with Tukey post-hoc testing or Kruskal-Wallis with Mann-Whitney test for numerical and Chi-square or Fisher’s exact test for categorical data.

## Data Availability

No new data were created or analyzed in this study.
